# Peripheral blood CD4^+^ T-lymphocyte count as a predictor of MRI findings in intracranial parenchymal tuberculomas

**DOI:** 10.3389/fneur.2026.1641464

**Published:** 2026-02-11

**Authors:** Xu-Wen Fu, Yan Bi, Qiu-Lan Shan, Yuan-Ying Li, Min Qi, Jia-Lu Wei, Hua He, Xiang Li

**Affiliations:** 1 Department of Clinical Pharmacy, Kunming Third People’s Hospital/Yunnan Clinical Medical Center for Infectious Diseases, Kunming, China; 2 Department of Radiology, The People’s Hospital of Lincang, Lincang, China; 3 Department of Radiology, Kunming Third People’s Hospital/Yunnan Clinical Medical Center for Infectious Diseases, Kunming, China; 4 Department of Biomedical Engineering, Kunming Third People’s Hospital/Yunnan Clinical Medical Center for Infectious Diseases, Kunming, China; 5 Department of Infectious Diseases, Kunming Third People’s Hospital/Yunnan Clinical Medical Center for Infectious Diseases, Kunming, China

**Keywords:** brain edema, CD4 lymphocyte count, central nervous system, T-lymphocytes, tuberculoma, tuberculosis

## Abstract

**Objective:**

This study aims to evaluate the association between peripheral blood CD4^+^ T-lymphocyte count and magnetic resonance imaging (MRI) features of intracranial parenchymal tuberculomas in patients diagnosed with hematogenous disseminated pulmonary tuberculosis.

**Methods:**

A retrospective analysis was conducted on patients diagnosed with hematogenous disseminated pulmonary tuberculosis accompanied by intracranial parenchymal tuberculomas. Patients were categorized into two groups based on MRI findings: those demonstrating perilesional edema (edematous type) and those without (non-edematous type). Demographic data, clinical symptoms, peripheral blood T-lymphocyte subsets, and additional MRI features were compared between the two groups.

**Results:**

Among 144 patients included in the analysis, 56 were classified into the edematous group and 88 into the non-edematous group. The frequency of headache was higher in the edematous group (60.7%) compared to the non-edematous group (40.9%). The median age was lower in the edematous group [27.0 years (IQR: 20.8, 42.0)] relative to the non-edematous group [33.5 years (IQR: 24.0, 51.0)]. A higher proportion of female patients was also observed in the edematous group (55.4%), compared to the non-edematous group (35.2%). Univariate analysis indicated that female sex, elevated peripheral blood CD3^+^ T-lymphocyte count, and elevated CD4^+^ T-lymphocyte count were significantly associated with the presence of perilesional edema [Odds ratio (OR) = 2.28 (95% Confidence Interval (CI): 1.15–4.522); OR = 1.001 (95% CI: 1.000–1.002); OR = 1.002 (95% CI: 1.000–1.003)], respectively. In multivariate analysis, elevated CD4^+^ T-lymphocyte count remained an independent predictor of perilesional edema [OR = 1.001 (95% CI: 1.000–1.002)]. On contrast-enhanced MRI, ring-enhancing lesions were more frequently observed in the edematous group (76.8%), compared to the non-edematous group (25%).

**Conclusion:**

In patients with hematogenous disseminated pulmonary tuberculosis and concurrent intracranial tuberculomas, higher peripheral CD4 + T-lymphocyte counts are independently associated with the presence of perilesional edema and a greater likelihood of ring-like enhancement on MRI.

## 1 Introduction

Tuberculosis (TB), caused by *Mycobacterium tuberculosis*, is a multisystem infectious disease that predominantly affects the lungs and remains a significant global public health concern. In 2023, approximately 10.8 million new TB cases were reported worldwide, including an estimated 741,000 new cases and 27,000 TB-related deaths in China (1).

Intracranial tuberculosis represents the most severe type of TB. Although its incidence is relatively low, the mortality rate remains high at approximately 25%, despite appropriate treatment (2). In developing countries, intracranial tuberculosis accounts for up to 50% of all TB-related deaths (3). Survivors frequently experience varying degrees of long-term neurological sequelae (4). Diagnosis requires a comprehensive evaluation of epidemiological context, clinical presentation, cerebrospinal fluid analysis, and neuroimaging studies, with magnetic resonance imaging (MRI) playing a particularly crucial role in detection and characterization (5).

Intracranial tuberculosis can be classified into meningeal tuberculosis, parenchymal tuberculosis, and mixed intracranial tuberculosis based on lesion location. Parenchymal tuberculosis involves infection within the cerebral, cerebellar, or brainstem parenchyma (6). Among these, intracranial tuberculomas are the most common form and may present with or without perilesional edema on MRI (7, 8). The presence of perilesional edema is likely associated with blood–brain-barrier (BBB) disruption, localized immune responses, and cytokine -mediated inflammation (9–11).

Tuberculomas associated with perilesional edema may exert a mass effect, leading to increased intracranial pressure (10). Increased intracranial pressure may impair cerebral perfusion, resulting in ischemic injury. This interaction between edema and ischemia can become a self-perpetuating cycle, potentially worsening clinical outcomes (12). Consequently, treatment for intracranial tuberculosis prioritize the reduction of intracranial pressure and enhancement of cerebral perfusion (13). In clinical settings—particularly those with limited imaging resources—tuberculomas without evident perilesional edema on non-contrast MRI may be underdiagnosed, highlighting the diagnostic significance of edema as a radiological marker (14).

T-lymphocytes play a significant role in the development and progression of intracranial tuberculosis. These cells traverse the BBB, accumulate in cerebrospinal fluid and parenchymal lesions, and contribute to local immune responses. Dysregulation of T-lymphocyte subsets has been associated with inflammatory activity and tissue damage (15). However, peripheral, cerebrospinal, and parenchymal T-lymphocyte counts and subsets are not linearly correlated due to the modulatory effects of the local cytokine microenvironment on lymphocyte trafficking and activation (16, 17). Direct assessment of lymphocyte subsets in cerebrospinal fluid and brain tissue remains technically challenging. Prior studies have demonstrated that peripheral CD4^+^ T-lymphocyte counts increase following treatment for intracranial tuberculosis and are associated with favorable prognosis (18). Nevertheless, the relationship between peripheral T-lymphocyte subsets count and MRI features of parenchymal tuberculomas has not been extensively investigated.

Accordingly, the present study retrospectively analyzed patients with hematogenous disseminated pulmonary tuberculosis complicated by intracranial parenchymal tuberculomas. The aim was to evaluate the relationship between peripheral blood T-lymphocyte subsets and MRI features, thereby improving the understanding of intracranial tuberculosis and providing preliminary imaging biomarkers for prognostic assessment.

## 2 Materials and methods

### 2.1 Study participants

This retrospective study included patients who were newly diagnosed with hematogenous disseminated pulmonary tuberculosis complicated by intracranial parenchymal tuberculosis at Kunming third people’s hospital between January 2020 and December 2023. Eligible patients had not received any prior antituberculosis therapy at the time of diagnosis. The study protocol was reviewed and approved by the ethics Committee of Kunming Third People’s hospital (ethics approval number: KSLL20230711001-01)

### 2.2 Inclusion and exclusion criteria

Inclusion criteria: Hematogenous disseminated pulmonary tuberculosis was diagnosed established in accordance with the “Health Industry Standard of the People’s Republic of China - Classification of Tuberculosis” (WS 196-2017) (19) and “Health Industry Standard of the People’s Republic of China - Diagnosis of Tuberculosis” (WS 288-2017) (20). The diagnostic criteria are as follows: Diagnosis is made through a comprehensive evaluation of clinical symptoms, signs, laboratory tests, and imaging studies. Pulmonary tuberculosis with hematogenous dissemination is divided into acute hematogenous pulmonary tuberculosis and subacute/chronic hematogenous pulmonary tuberculosis. In the acute form, imaging shows uniformly distributed or evenly distributed miliary nodules of consistent size and density throughout both lungs. In the subacute/chronic form, the lesions are located in the upper and middle parts of both lungs, with varying sizes and densities, and some may show coalescence. Intracranial tuberculosis was diagnosed based on the “2019 Chinese Guidelines for the Diagnosis and Treatment of Central Nervous System Tuberculosis” (21). The diagnostic criteria are as follows: Cases confirmed with central nervous system tuberculosis through positive microbiological or nucleic acid amplification tests for *Mycobacterium tuberculosis* are selected. Alternatively, highly suspected cases are diagnosed based on a scoring system that incorporates clinical manifestations, cerebrospinal fluid findings, neuroimaging findings, and other evidence of tuberculosis. Parenchymal tuberculosis was confirmed by imaging as defined by the “Expert Consensus on Imaging classification for Intracranial Tuberculosis,” published by the Chinese Medical Association in 2015 (6). The diagnostic criteria are as follows: On cranial MRI, the presence of tuberculomas within the brain parenchyma of the cerebrum, cerebellum, or brainstem is demonstrated. These tuberculomas appear as lesions of varying sizes with round, oval, or irregular shapes. The signal characteristics of the tuberculomas differ based on the presence or absence of central caseous necrosis or liquefactive necrosis. Perituberculoma brain edema may or may not be present.

Exclusion criteria: Patients were excluded if they had incomplete clinical or imaging data; concurrent HIV infection; absence of parenchymal tuberculomas; or evidence of other intracranial infections or brain tumors.

### 2.3 Clinical data

Clinical data were collected from the hospital’s electronic medical record system. Collected variables included demographic characteristics (age, sex, body mass index [BMI]), presence or absence of extrapulmonary tuberculosis, and reported clinical symptoms. Symptoms assessed were fever, night sweats, weight loss, cough, headache, vomiting, and altered consciousness.

### 2.4 Laboratory tests

Peripheral blood T-lymphocyte subset counts were obtained from samples collected within 24 h of hospital admission. Testing was performed using a Beckman Flow Cytometer (Beckman Coulter Inc., United States). A total of 200 μL of whole blood was used for each analysis.

### 2.5 Imaging examinations

Initial MRI of the brain was performed within 48 h of admission. Imaging was conducted using either the United Imaging uMR588 or uMR780 scanner (United Imaging, Shanghai, China). Both non-contrast and contrast-enhanced MRI sequences were acquired. Non-contrast scans included T2-weighted imaging (T2WI), T2 fluid-attenuated inversion recovery (T2FLAIR), and diffusion-weighted imaging (DWI), with a slice thickness of 5 mm. High-resolution sagittal T1-weighted imaging (T1WI) was conducted using isotropic voxel volumes of 1 mm × 1 mm × 1 mm, with subsequent multiplanar reconstructions in the transverse and coronal planes (slice thickness: 1 mm). Contrast-enhanced scans included T2FLAIR sequences (slice thickness: 5 mm), and enhanced T1WI with isotropic voxel volumes of 1 mm × 1 mm × 1 mm, followed by multiplanar reconstructions in the transverse and coronal planes (slice thickness: 1 mm). The contrast medium used was Gadoteric Acid Meglumine (Jiangsu Hengrui Pharmaceuticals Co., Ltd., Jiangsu, China), administered at a dosage of 0.2 mL/kg.

### 2.6 Imaging analysis and case grouping

Imaging data were retrieved from the hospital’s Picture Archiving and Communication System (PACS) and reviewed using the Radiant DICOM Viewer (Poland). Imaging features assessed included the number and enhancement patterns of parenchymal tuberculomas, presence or absence of perilesional edema, coexisting meningeal tuberculosis, cerebral infarction, and hydrocephalus. Lesions were classified as solitary or multiple based on lesion count. Meningeal involvement was defined as thickening of the basal cisterns, Sylvian fissure cistern, or cerebral convexity meninges. The diagnosis of hydrocephalus was based on the “Chinese Expert Consensus on Standardized Treatment for Hydrocephalus” by the Chinese Congress of Neurological Surgeons in 2013 (22) and the Evans Index criterion (23). Parenchymal tuberculomas were classified by contrast enhancement patterns into two types: enhancement homogeneous and ring-like enhancement. Images were reviewed independently and in a blinded manner by radiologists (with intermediate or senior professional titles) specializing in infectious disease imaging. In cases of disagreement, a third senior radiologist reviewed the images to ensure a final consensus.

**Figure 1:**
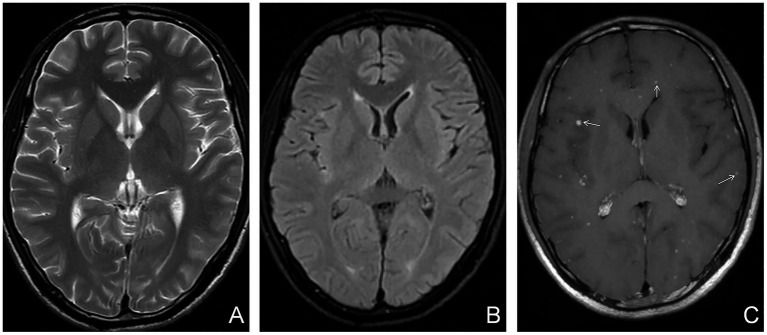
Non-edematous parenchymal tuberculoma. **(A)** Axial T2WI showing no abnormalities. **(B)** Axial T2-FLAIR image also showing no abnormal findings. **(C)** Axial contrast-enhanced T1WI image showing multiple homogeneously enhancing nodular lesions (white arrows) in the brain parenchyma, without hypointense perilesional edema.

**Figure 2:**
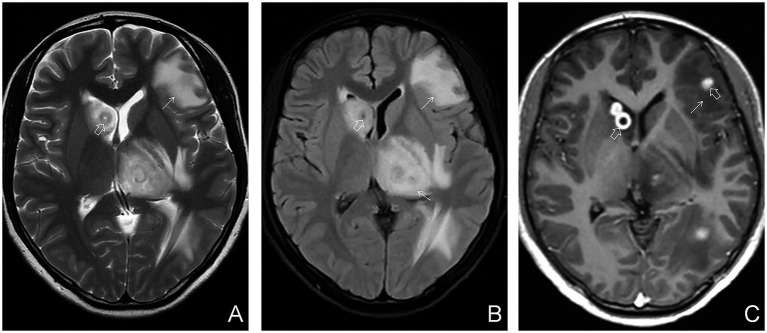
Edematous parenchymal tuberculoma. **(A)** Axial T2WI plain scan showing multiple patchy hyperintense lesions in the brain parenchyma, indicating edema (white arrows), with centrally located heterogeneous lesions suggestive of tuberculomas (hollow arrows). **(B)** Axial T2FLAIR plain scan showing similar edema and central tuberculomas (white and hollow arrows, respectively). **(C)** Axial contrast-enhanced T1WI demonstrating both ring-like and homogeneously enhancing nodules (hollow arrows), consistent with tuberculomas, surrounded by patchy hypointense areas indicative of perilesional edema (white arrows).

Based on the presence or absence of perilesional edema, tuberculomas were further categorized into edematous and non-edematous types. Perilesional edema was identified by characteristic imaging features: patchy hypointensity on T1WI, hyperintensity on T2WI and T2FLAIR, with no restricted diffusion on DWI. On enhanced T1WI, edema did not exhibit enhancement, whereas the central tuberculoma demonstrated either homogeneous or ring-like enhancement (Figures 1, 2). A case–control design was employed to compare demographic characteristics, clinical symptoms, peripheral blood T-lymphocyte subset counts, and imaging findings between the edematous and non-edematous groups.

### 2.7 Statistical methods

All statistical analyses were performed using SPSS software, version 26.0. Quantitative variables were first assessed for normality. Variables with a normal distribution were expressed as mean ± standard deviation (*x̄* ± *s*), and comparisons between two groups were conducted using the independent samples *t*-tests. For non- normally distributed, results were expressed as median and interquartile range [M (Q1, Q3)], and group comparisons were made using the Mann–Whitney U test. A two-sided *p*-value < 0.05 was considered statistically significant. Categorical variables were expressed as frequencies and percentages, and intergroup comparisons were performed using the chi-squared test. For univariate logistic regression analysis, variables with *p*-values < 0.10 were entered into a multivariate logistic regression model to identify independent predictors.

## 3 Results

### 3.1 Case grouping

A total of 192 patients were initially identified, all newly diagnosed with hematogenous disseminated pulmonary tuberculosis complicated by parenchymal tuberculosis at Kunming Third People’s Hospital. Of these, 38 patients were excluded due to incomplete clinical or imaging data, 9 due to co-infection with HIV, and 1 due to tuberculous encephalitis. Ultimately, 144 patients met the inclusion criteria and were included in the final analysis. Among these, 82 (56.9%) were male and 62 (43.1%) were female, with a median age of 31.0 years (IQR: 22.0, 48.8 years).

Based on cranial MRI findings, participants were categorized into edematous and non-edematous types according to the presence or absence of perilesional edema surrounding the parenchymal tuberculomas, respectively. In all, 56 patients (38.9%) were classified as having the edematous type, while 88 (61.1%) were classified as non-edematous.

### 3.2 Clinical symptoms

Among the 144 patients included in the study, fever was reported in 113 cases (78.5%), weight loss in 47 (32.6%), night sweats in 22 (15.3%), cough in 85 (59.0%), headache in 70 (48.6%), vomiting in 48 (33.3%), and altered consciousness in 18 (12.5%). The incidence of headache was significantly higher in the edematous group (60.7%) compared to the non-edematous group (40.9%), with a statistically significant difference (*p* < 0.05) (see Table 1).

**Table 1:** Clinical symptoms of patients in the two groups.

Symptom	All patients (*n* = 144)	Non-edematous type (*n* = 88)	Edematous type (*n* = 56)	Value of test	*p*-value
Fever	113 (78.5%)	73 (83.0%)	40 (71.4%)	2.05	0.152
Weight loss	47 (32.6%)	30 (34.1%)	17 (30.4%)	0.08	0.777
Night sweats	22 (15.3%)	12 (13.6%)	10 (17.9%)	0.20	0.654
Coughing	85 (59.0%)	53 (60.2%)	32 (57.1%)	0.04	0.847
Headache	70 (48.6%)	36 (40.9%)	34 (60.7%)	4.61	0.032
Vomiting	48 (33.3%)	25 (28.4%)	23 (41.1%)	1.93	0.165
Disturbance of consciousness	18 (12.5%)	10 (11.4%)	8 (14.3%)	0.07	0.796

### 3.3 General data and T-lymphocyte subsets

Of the 144 cases, 56 patients (38%) were classified into the edematous group and 88 (61.1%) into the non-edematous group. In the edematous group, there were 25 males (44.6%) and 31 females (55.4%), with a median age of 27.0 years (IQR: 20.8, 42.0) and a median BMI of 18.73 kg/m^2^ (IQR: 16.58, 21.18). Additionally, 28 cases (50%) presented with extrapulmonary tuberculosis besides central nervous system involvement. In the non-edematous group, there were 57 males (64.8%) and 31 females (35.2%), with a median age of 33.5 years (IQR: 24.0, 51.0) and a median BMI of 20.28 kg/m^2^ (17.39, 22.12). Furthermore, 45 patients (51.1%) presented with extrapulmonary tuberculosis in addition to central nervous system involvement.

The median age was significantly lower in the edematous group, and the proportion of female patients was higher in the edematous group than in the non-edematous group (all *p*-values < 0.05).

Median peripheral blood counts of CD3^+^ T-lymphocytes, CD4^+^ T-lymphocytes, and CD8^+^ T-lymphocytes were all significantly higher in the edematous type than in the non-edematous group, with statistically significant differences (all *p* < 0.05, Table 2). Univariate logistic regression analysis identified female sex, elevated peripheral CD3^+^ T-lymphocyte count, and elevated peripheral CD4^+^ T-lymphocyte count as factors associated with an increased likelihood of perilesional edema in parenchymal tuberculomas: Female sex: OR = 2.28, 95% CI: 1.15–4.522; CD3^+^ T-lymphocyte count: OR = 1.001; 95% CI: 1.000–1.002; and CD4 + T-lymphocytes count: OR = 1.002; 95% CI: 1.000–1.003. Multivariate logistic regression analysis demonstrated that elevated CD4^+^ T-lymphocyte count remained an independent predictor of perilesional edema (OR = 1.001; 95% CI: 1.000–1.002) (Table 3).

**Table 2:** Comparative analysis of baseline characteristics between the two groups.

Variable	All patients (*n* = 144)	Non-edematous type (*n* = 88)	Edematous type (*n* = 56)	Value of test	All patients (*n* = 144)
Age (year)	31.0 (22.0, 48.8)	33.5 (24.0, 51.0)	27.0 (20.8, 42.0)	−2.185	0.029
Sex (male)	82 (56.9%)	57 (64.8%)	25 (44.6%)	4.86	0.027
CD3+	705.00 (430.77, 1103.00)	615.50 (354.00, 1021.00)	907.00 (550.00, 1372.00)	2.959	0.003
CD4+	403.00 (221.50, 609.75)	346.00 (176.15, 511.88)	517.00 (282.25, 750.50)	3.035	0.002
CD8+	276.50 (167.75, 448.00)	244.00 (152.50, 396.10)	336.50 (220.00, 551.00)	2.512	0.012
BMI	19.68 (16.91, 22.03)	20.28 (17.39, 22.12)	18.73 (16.58, 21.18)	−1.758	0.079
EPTB	73 (50.7%)	45 (51.1%)	28 (50.0%)	0.018	0.894

EPTB, Extrapulmonary tuberculosis; BMI, Body mass index = Body mass (Kg)/Height (m)^2^.

**Table 3:** Univariate and multivariate logistic regression analysis.

Variable	Univariate analysis						Multivariate analysis					
	*B*	SE	OR	95%CI	*Z*	*P*	*B*	SE	OR	95%CI	*Z*	*P*
Age	−0.02	0.01037	0.98	0.961–1	−1.916	0.055						
Female	0.824	0.34937	2.28	1.15–4.522	2.359	0.018	0.59	0.36892	1.805	0.876–3.719	1.601	0.109
CD3+	0.001	0.00034	1.001	1–1.002	2.523	0.012						
CD4+	0.002	0.00055	1.002	1–1.003	2.714	0.007	0.001	0.00057	1.001	1–1.002	2.162	0.031
CD8+	0.001	0.00076	1.001	1–1.003	1.704	0.088						

### 3.4 MRI imaging features

Regarding the number of lesions, multiple brain parenchymal tuberculomas were observed in 138 cases (95.8%), while solitary lesions were observed in 6 cases (4.2%). Parenchymal tuberculomas typically exhibited isointense or slightly hypointense signals on T1WI, and isointense or heterogeneous signals on T2WI. Following contrast administration, lesions demonstrated either homogeneous or ring-like enhancement.

**Figure 3:**
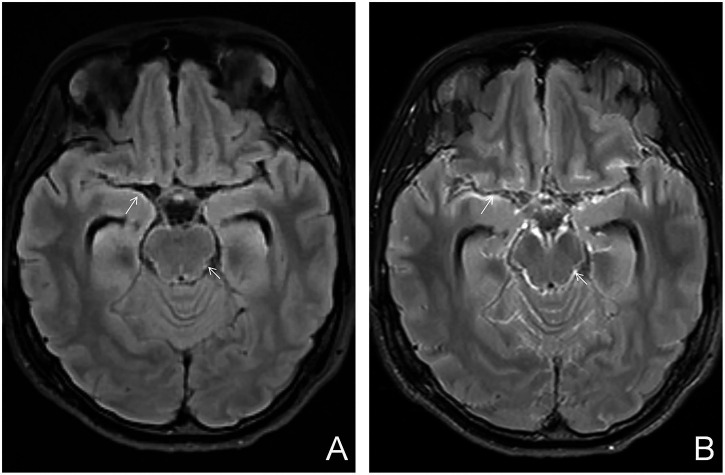
Parenchymal tuberculoma with concurrent meningeal tuberculosis. **(A)** Axial T2-FLAIR image showing mild meningial thickening in the basal cistern and ambient cistern (white arrow), indicating meningeal tuberculosis; **(B)** Axial contrast-enhanced T2-FLAIR image showing marked enhancement of the thickened meninges in the basal cistern and ambient cistern (white arrow).

**Figure 4:**
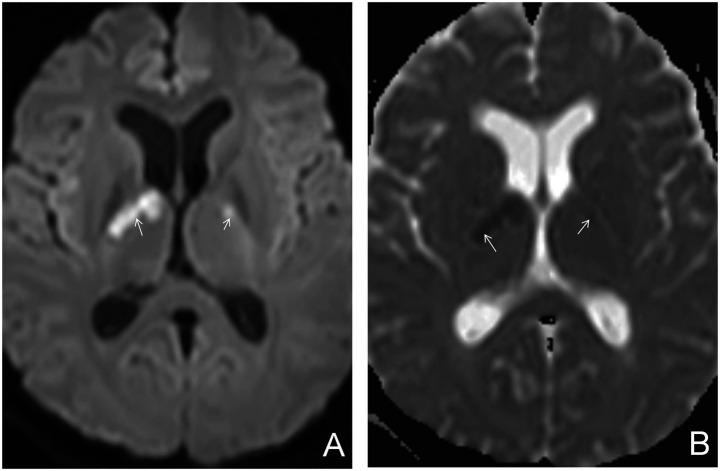
Parenchymal tuberculous granulomas with concurrent cerebral infarction. **(A)** DWI (*B* = 1,000) showing multiple hyperintense lesions in the bilateral basal ganglia (white arrows), indicating acute cerebral infarction. **(B)** ADC map showing hypointensity in the bilateral basal ganglia (white arrows).

**Figure 5:**
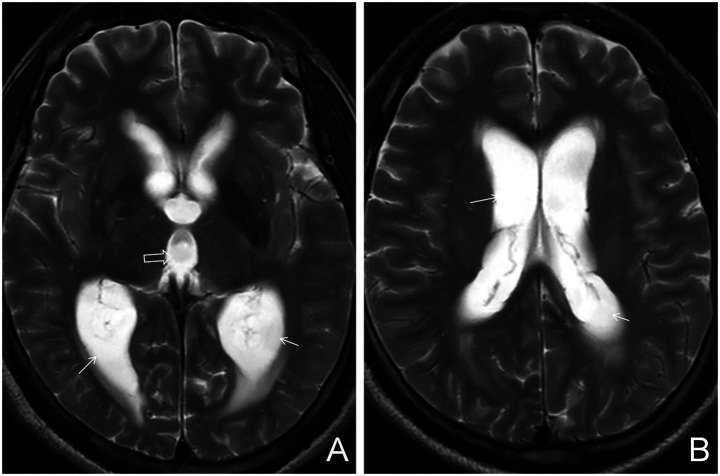
Parenchymal tuberculoma with concurrent hydrocephalus. **(A)** Axial T2WI showing enlargement of the trigones of both lateral ventricles (white arrows) and the third ventricle (hollow arrow), indicating hydrocephalus. **(B)** Axial T2WI showing enlargement of the anterior and posterior horns of both lateral ventricles (white arrows), indicating hydrocephalus.

Additional MRI findings included coexisting meningeal tuberculosis, cerebral infarction, and hydrocephalus. Meningeal tuberculosis was characterized by thickening and contrast enhancement of the basal cisterns, ambient cisterns, Sylvian fissures, and cerebral convexity meninges (Figure 3). Cerebral infarction presented as hyperintense signals on DWI and hypointense signals on apparent diffusion coefficient (ADC) maps (Figure 4). Hydrocephalus was characterized by ventricular enlargement with rounded margins and an EVANS index > 0.3 (Figure 5).

Among the 144 cases, ring-like enhancement was observed in 65 cases (45.1%), while homogeneous enhancement was observed in 79 cases (54.9%). Concurrent meningeal tuberculosis was identified in 52 cases (36.1%), cerebral infarction in 20 cases (13.9%), and hydrocephalus in 10 cases (6.9%). The proportion of ring-like enhancement was significantly higher in the edematous group (76.8%) compared to the non-edematous group (25.0%), with the difference reaching statistical significance (*p* < 0.05, Table 4).

**Table 4:** MRI features of parenchymal tuberculomas in the edematous and non-edematous groups.

Imaging signs	All patients (*n* = 144)	Non-edematous type (*n* = 88)	Edematous type (*n* = 56)	Value of test	*p*-value
Multiple lesions	138 (95.8%)	85 (96.6%)	53 (94.6%)	NA	0.678
Ring-like enhancement	65 (45.1%)	22 (25.0%)	43 (76.8%)	35.00	<0.001
Cerebral infarction	20 (13.9%)	9 (10.2%)	11 (19.6%)	1.81	0.178
Meningeal tuberculosis	52 (36.1%)	30 (34.1%)	22 (39.3%)	0.21	0.649
Hydrocephalus	10 (6.9%)	7 (8.0%)	3 (5.4%)	NA	0.741

*Using Fisher’s Precision Testing.

## 4 Discussion

This study examined patients diagnosed with hematogenous disseminated pulmonary tuberculosis complicated by parenchymal tuberculomas to investigate the association between perilesional edema and peripheral blood T-lymphocyte subsets. Previous studies have reported that meningeal tuberculosis occurs more frequently than parenchymal tuberculosis, potentially due to the higher density of astrocytes and resident immune cells in the meninges (24). However, in the present study, more than half of the patients with parenchymal tuberculomas did not exhibit concurrent meningeal involvement. This observation suggests that meningeal and parenchymal tuberculosis may not simply represent spatial variations of the same disease process but could reflect distinct stages or pathophysiological mechanisms within the spectrum of intracranial tuberculosis. Further mechanistic studies are warranted to delineate these differences. By focusing exclusively on patients with parenchymal tuberculomas, this study aimed to enhance the understanding of how peripheral immune responses, particularly T-lymphocyte subsets, relate to intracerebral inflammatory processes observable on MRI. These insights may have clinical implications for evaluating the inflammatory status and treatment response, particularly with regard to the use of adjunctive glucocorticoid therapy. Radiological features such as perilesional edema may serve as potential imaging biomarkers of immunological activity, thus contributing to more tailored treatment strategies in intracranial tuberculosis.

With respect to clinical symptoms, most patients in this study presented with systemic symptoms such as fever and cough, consistent with the diagnosis of hematogenous disseminated pulmonary tuberculosis. In contrast, neurological symptoms, including headache, vomiting, and altered consciousness, were less frequent. This may be attributed to the localization of the tuberculomas within the brain parenchyma and the absence of concurrent meningeal involvement in a substantial proportion of cases. Without meningeal inflammation, signs of meningeal irritation such as headache and vomiting are less prominent. However, patients with perilesional edema experienced more frequent headaches than those without edema. This is likely due to the localized mass effect and resulting elevation in intracranial pressure. The relative absence of central nervous system symptoms in some cases, particularly in the non-edematous group, may contribute underdiagnosis or delayed diagnosis in clinical practice.

The clinical and radiological manifestations of intracranial tuberculosis are influenced by both the virulence of *M. tuberculosis* and the host immune response. Neutrophils contribute to the innate immune response, whereas lymphocytes are central to adaptive immunity. B-lymphocytes represent a small proportion of immune cells in the cerebrospinal fluid during intracranial tuberculosis and appear to play a limited role in local immune defense (16). In contrast, T-lymphocytes, especially CD4^+^ and CD8^+^ subsets, are crucial to the immunological containment of intracranial tuberculosis. CD4^+^ T cells enhance macrophage activation through the secretion of interferon-γ (IFN-γ), enhancing intracellular bacterial clearance, while CD8^+^ T cells exert cytotoxic effects contributing to pathogen elimination (25). Additionally, T-lymphocytes cells play a central role in the formation and maintenance of tuberculous granulomas, which serve to limit dissemination. However, excessive immune activation may also induce tissue necrosis and perilesional edema (26). In the present study, patients with parenchymal tuberculomas accompanied by perilesional edema were more frequently female and had a lower median age compared to those without edema. This pattern may reflect a more robust immune response and the immunomodulatory influence of estrogen in females, which has been associated with enhanced Th1-type immune activity and increased pro-inflammatory cytokine production (27).

The observed association between elevated peripheral CD4^+^ T-lymphocyte counts and the presence of perilesional edema on MRI raises the question of how systemic immune markers relate to local inflammatory processes within the CNS. While direct measurement of T-cell subsets in brain tissue is not feasible in clinical practice, peripheral CD4^+^ counts may serve as an accessible surrogate reflecting the overall cellular immune status. In tuberculosis, CD4^+^ T cells are pivotal in orchestrating the immune response through cytokine secretion, such as IFN-γ, which activates macrophages and enhances granuloma formation (28). However, the relationship between peripheral counts and intraparenchymal inflammation is complex and non-linear, influenced by the local cytokine microenvironment and the integrity of the BBB (29). A study suggests that during active CNS tuberculosis, there is a coordinated trafficking of activated T cells across the BBB, where they contribute to the local immune reaction (30). The perilesional edema observed on MRI likely represents the culmination of this localized inflammatory cascade, involving cytokine-mediated vasogenic edema and cellular infiltration. Therefore, a higher peripheral CD4^+^ count in immunocompetent patients may indicate a more vigorous systemic immune response that, upon trafficking into the CNS, contributes to a more intense local inflammation visible as edema on imaging. This conceptual link aligns with findings from animal models and clinical studies where Th1-type immune responses are associated with both protective immunity and immunopathology in CNS infections. Future studies correlating peripheral immune markers with simultaneous cerebrospinal fluid cytokine profiles and advanced neuroimaging parameters would be valuable to further delineate this systemic-to-central inflammatory axis.

It is noteworthy that while the OR for the association between CD4^+^ T-lymphocyte count and edema in this study (1.001) is close to 1, its statistical significance and clinical relevance must be interpreted in the context of the variable’s unit of measurement and the study design. Since CD4^+^ count is a continuous variable (unit: cells/μL), an OR of 1.001 indicates that for every increase of 1 cell/μL, the odds of perilesional edema increase by approximately 0.1%. In clinical practice, CD4^+^ counts typically fluctuate on the order of tens to hundreds of cells/μL, and the cumulative effect on edema risk becomes more pronounced (e.g., an increase of 100 cells/μL corresponds to an OR of approximately 1.105). Therefore, the present findings support a positive dose–response relationship between peripheral CD4^+^ T-lymphocyte levels and intracranial inflammatory responses, rather than providing a threshold for individual diagnosis. This observation suggests that among patients with hematogenously disseminated pulmonary tuberculosis, a higher systemic cellular immune status may be associated with more prominent inflammatory imaging manifestations within the central nervous system, which could help identify patient subgroups that may benefit from more proactive neuroimaging evaluation and adjunctive anti-inflammatory therapy.

With respect to MRI features, parenchymal tuberculomas were predominantly observed as multiple lesions, which is consistent with the underlying pathophysiology of hematogenous dissemination of *M. tuberculosis* in the central nervous system (31). On contrast-enhanced MRI, parenchymal tuberculomas typically exhibited either homogeneous or ring-like enhancement. These enhancement patterns correspond to distinct histopathological stages of tuberculoma development. Based on histopathology, parenchymal tuberculomas are generally classified into non-necrotizing granulomas, necrotizing granulomas, and tuberculous abscesses (24). Homogeneous enhancement is commonly associated with non-necrotizing granulomas. In contrast, ring-like enhancement is characteristic of necrotizing granulomas or abscesses, where the central necrotic core fails to enhance due to lack of perfusion, while the surrounding viable tissue demonstrates marked contrast uptake (8). Although MRI cannot be used to reliably differentiate between necrotizing granulomas and tuberculous abscesses, both are associated with heightened local immune activity.

Granulomas containing central necrosis are surrounded by dense infiltrates of T lymphocytes and macrophages, which release cytokines such as tumor necrosis factor-alpha (TNF-α) and interleukin-6 (IL-6). These cytokines contribute to local immune activation and development of perilesional edema (32). In the present study, ring-like enhancement was significantly more common in tuberculomas with perilesional edema compared to those without, supporting a link between necrotic pathology and the occurrence of edema. Moreover, peripheral CD4^+^ T-lymphocyte count was identified as an independent predictor perilesional edema. It should be noted, however, that this cross-sectional study design only demonstrates an association between higher CD4^+^ T-cell counts and edema presence, and does not establish a causal relationship. Perilesional edema may also serve as a marker of a more advanced or active disease phase, rather than being directly driven by systemic CD4^+^ T-cell levels.

A higher CD4^+^ T-cell count may indicate stronger cellular immune function. The presence of perilesional edema in parenchymal tuberculomas is attributed to intense inflammatory reactions around the granuloma, which aligns with findings indicating robust cellular immunity characterized by CD4^+^ T cells predominance in patients. However, previous studies have reported that perilesional edema is more commonly observed in patients with HIV infection, despite their impaired cellular immunity (2). This apparent contradiction suggests that perilesional edema may arise through distinct immunopathological mechanisms in different immunological contexts. In HIV-infected individuals with low CD4^+^ counts, edema might result from dysregulated immune responses, cytokine storm, or secondary infections, rather than CD4^+^ T-cell-mediated immunity. Conversely, in immunocompetent patients, edema may reflect a more coordinated but potentially excessive cellular immune response. To date, no controlled studies have stratified edema severity in relation to peripheral or cerebrospinal immune profiles, highlighting the need for further mechanistic investigation. Therefore, the precise immunopathological mechanisms linking systemic immune markers to the degree of cerebral edema remain unclear. Future studies are needed to characterize the precise relationship between the severity of perilesional edema and specific immune cell subsets, as well as to correlate imaging findings with cytokine levels in both serum and cerebrospinal fluid.

The imaging features of parenchymal tuberculomas lack specificity. In particular, ring-like enhancement accompanied by perilesional edema can mimic appearances of brain metastases or other intracranial infections caused by different pathogens. Additionally, intracranial tuberculosis may be complicated by hydrocephalus and cerebral infarction, both of which are associated with significant morbidity. Hydrocephalus may impair cerebral perfusion, while cerebral infarction can result in irreversible neurological deficits. Therefore, the differential diagnosis of intracranial tuberculosis requires careful consideration.

This study has several limitations. First, the retrospective design inherently carries a risk of selection bias and limits the ability to establish causal inferences between CD4^+^ T-lymphocyte counts and MRI findings. Second, stratified analyses of CD4^+^ and CD8^+^ T-lymphocyte counts were not conducted, precluding evaluation of threshold effects or dose–response relationships between T-cell levels and imaging features. Meanwhile, due to the moderate sample size, we were also unable to perform stratified analyses (e.g., by sex or age subgroups) to explore potential effect modification by demographic factors. Future larger studies are warranted to examine whether the association between peripheral CD4^+^ T-lymphocyte counts and imaging features varies across different patient subgroups. Third, no follow-up was conducted to assess responses to antituberculosis therapy. Furthermore, this study primarily relied on the qualitative grouping of edema based on its “presence or absence” and did not further quantify the degree of edema or lesion size for correlation analysis with peripheral immune markers. This is mainly due to the current lack of a widely accepted imaging grading standard for perilesional edema in intracranial tuberculomas, and the absence of systematically collected precise lesion dimension data in this retrospective design. Future prospective studies are warranted to establish standardized imaging assessment parameters to more precisely elucidate the quantitative relationships between immune cell counts and the pathological manifestations of intracranial tuberculosis. Fourth, due to the integrated tuberculosis management system in China where follow-up care is typically transferred to local community health centers, we were unable to collect systematic data on treatment outcomes or longitudinal imaging changes. Consequently, the prognostic implications of our findings remain unclear. The lack of outcome data also limited exploration of prognostic implications associated with T-lymphocyte subsets or MRI findings. Additionally, all variables with *p* < 0.10 in univariate analysis were entered simultaneously into the multivariate model, rather than using a stepwise selection procedure. While this approach was chosen for stability given our sample size, it does not automatically account for potential multicollinearity among predictors. In particular, we did not formally assess the collinearity between CD3^+^ and CD4^+^ T-lymphocyte counts, which are biologically correlated. Nevertheless, the multivariate result, in which the CD4^+^ subset remained significant while the total CD3^+^ count did not, underscores the more specific role of CD4^+^ T cells in the immunopathology reflected by perilesional edema.

In summary, disseminated pulmonary tuberculosis complicated by parenchymal tuberculomas may present with perilesional edema, particularly in individuals with elevated peripheral CD4^+^ T-lymphocyte counts. Female sex and younger age were associated with a higher incidence of edema and ring-enhancing lesions, which also correlated with more frequent headache symptoms. In contrast, male patients with lower CD4^+^ T-cell counts may present without perilesional edema and may therefore be at a greater risk for underdiagnosis, especially when contrast-enhanced MRI is not performed. These findings underscore the importance of gadolinium-enhanced MRI in the accurate detection of intracranial tuberculomas and suggest a potential role for peripheral immune markers in guiding clinical evaluation.

## Data Availability

The original contributions presented in the study are included in the article/supplementary material, further inquiries can be directed to the corresponding authors.

## References

[1] World Health Organization. Global tuberculosis report 2024. Available online at: https://www.who.int/publications/i/item/9789240101531 (Accessed October 29, 2024).

[2] Wang MG Luo L Zhang Y Liu X Liu L He J-Q. Treatment outcomes of tuberculous meningitis in adults: a systematic review and meta-analysis. BMC Pulm Med. (2019) 19:200. doi: 10.1186/s12890-019-0966-8, 31694599 PMC6833188

[3] Wasay M Farooq S Khowaja ZA Bawa ZA Ali SM Awan S . Cerebral infarction and tuberculoma in central nervous system tuberculosis: frequency and prognostic implications. J Neurol Neurosurg Psychiatry. (2014) 85:1260–4. doi: 10.1136/jnnp-2013-307178, 24623792

[4] Kalita J Misra UK Singh VK Pandey PC Thomas J. Inclusion of mechanical ventilation in severity staging of tuberculous meningitis improves outcome prediction. Am J Trop Med Hyg. (2020) 103:689–95. doi: 10.4269/ajtmh.20-0077, 32458779 PMC7410456

[5] Marais S Thwaites G Schoeman JF Török ME Misra UK Prasad K . Tuberculous meningitis: a uniform case definition for use in clinical research. Lancet Infect Dis. (2010) 10:803–12. doi: 10.1016/S1473-3099(10)70138-9, 20822958

[6] Chinese Tuberculosis Association. Imaging classification expert consensus of intracranial tuberculosis. Chin J Tuberc Respir Dis. (2015) 38:805–9. doi: 10.3760/cma.j.issn.1001-0939.2015.11.003

[7] Dahal P Parajuli S. Magnetic resonance imaging findings in central nervous system tuberculosis: a pictorial review. Heliyon. (2024) 10:e29779. doi: 10.1016/j.heliyon.2024.e29779, 38699716 PMC11063446

[8] Khatri GD Krishnan V Antil N Saigal G. Magnetic resonance imaging spectrum of intracranial tubercular lesions: one disease, many faces. Pol J Radiol. (2018) 83:e524–35. doi: 10.5114/pjr.2018.81408, 30800191 PMC6384409

[9] Xiao X Li Q Ju Y. Giant central nervous system tuberculoma in pediatric patients: surgical case series. Childs Nerv Syst. (2021) 37:2935–41. doi: 10.1007/s00381-021-05091-1, 33675392 PMC8423696

[10] Abbasi F Ozer M Juneja K Goksu SY Mobarekah BJ Whitman MS. Intracranial Tuberculoma mimicking Neurosarcoidosis: a clinical challenge. Infect Dis Rep. (2021) 13:181–6. doi: 10.3390/idr13010020, 33804334 PMC7930965

[11] Abu-Abaa M Arshad H Ali D Abdulsahib A. Meningeal tuberculoma masquerading as a brain tumor: a case report. Cureus. (2022) 14:e30804. doi: 10.7759/cureus.30804, 36457642 PMC9705070

[12] Zadka Y Doron O Rosenthal G Barnea O. Mechanisms of reduced cerebral blood flow in cerebral edema and elevated intracranial pressure. J Appl Physiol. (2023) 134:444–54. doi: 10.1152/japplphysiol.00287.2022, 36603049

[13] Donovan J Figaji A Imran D Phu NH Rohlwink U Thwaites GE. The neurocritical care of tuberculous meningitis. Lancet Neurol. (2019) 18:771–83. doi: 10.1016/S1474-4422(19)30154-1, 31109897

[14] De Luca F Kits A Martin MD Aspelin Å Kvist O Österman Y . Elective one-minute full brain multi-contrast MRI versus brain CT in pediatric patients: a prospective feasibility study. BMC Med Imaging. (2024) 24:23. doi: 10.1186/s12880-024-01196-6, 38267889 PMC10809606

[15] Chaudhary N Gupta BK Poudel A Kafle M Singh N Chaudhary HP. Stroke in a child with pulmonary tuberculosis and pleural effusion-an important clue for the diagnosis of disseminated central nervous system tuberculosis. Clin Case Rep. (2023) 11:e6945. doi: 10.1002/ccr3.6945, 36789297 PMC9914082

[16] Yu N Zhang QQ Zhang K Xie Y Zhu HQ Lin XJ . Comparative study of CD4 and CD45RO T cells and CD20 B cells in cerebrospinal fluid of syphilitic meningitis and tuberculous meningitis patients. APMIS. (2016) 124:764–9. doi: 10.1111/apm.12572, 27467195

[17] Tomalka J Sharma A Smith A Smith AGC Avaliani T Gujabidze M . Combined cerebrospinal fluid metabolomic and cytokine profiling in tuberculosis meningitis reveals robust and prolonged changes in immunometabolic networks. Tuberculosis (Edinb). (2024) 144:102462. doi: 10.1016/j.tube.2023.102462, 38070353 PMC10842779

[18] He H Zhang YL Li Y Huang Y Li X Xu J . Efficacy and prognostic value of peripheral blood CD4(+) T cells and serum IL-6 and IL-8 in tuberculous meningitis. Heliyon. (2024) 10:e31641. doi: 10.1016/j.heliyon.2024.e31641, 38845916 PMC11154195

[19] People’s Republic of China state health and Family Planning Commission. Tuberculosis classification(WS196—2017)[J/CD]. Electronic Journal of Emerging Infectious Diseases. (2018) 3:188–90.

[20] People’s Republic of China state health and Family Planning Commission. Diagnosis for pulmonary tuberculosis(WS196—2017)[J/CD]. Electronic Journal of Emerging Infectious Diseases. (2018) 3:59–61.

[21] Chinese Tuberculosis Association, Tuberculous Meningitis Committee. Guidelines for diagnosis and treatment of central nervous system tuberculosis. Chin J Infect Dis. (2020) 38:400–8. doi: 10.3760/cma.j.cn311365-20200606-00645

[22] Chinese Neurosurgical Society. Chinese expert consensus on standardized treatment of hydrocephalus. Chin J Neurosurg. (2013) 29:634–7. doi: 10.3760/cma.j.issn.1001-2346.2013.06.035

[23] Raut T Garg RK Jain A Verma R Singh MK Malhotra HS . Hydrocephalus in tuberculous meningitis: incidence, its predictive factors and impact on the prognosis. J Infect. (2013) 66:330–7. doi: 10.1016/j.jinf.2012.12.009, 23291048

[24] Zaharie SD Franken DJ van der Kuip M van Elsland S de Bakker BS Hagoort J . The immunological architecture of granulomatous inflammation in central nervous system tuberculosis. Tuberculosis (Edinb). (2020) 125:102016. doi: 10.1016/j.tube.2020.102016 33137697

[25] Shridhar A Garg RK Rizvi I Jain M Ali W Malhotra HS . Prevalence of primary immunodeficiency syndromes in tuberculous meningitis: a case-control study. J Infect Public Health. (2022) 15:29–35. doi: 10.1016/j.jiph.2021.11.019, 34883295

[26] Ma Q Chen J Kong X Zeng Y Chen Z Liu H . Interactions between CNS and immune cells in tuberculous meningitis. Front Immunol. (2024) 15:1326859. doi: 10.3389/fimmu.2024.1326859 38361935 PMC10867975

[27] Gupta M Srikrishna G Klein SL Bishai WR. Genetic and hormonal mechanisms underlying sex-specific immune responses in tuberculosis. Trends Immunol. (2022) 43:640–56. doi: 10.1016/j.it.2022.06.004, 35842266 PMC9344469

[28] Bromley JD Ganchua SKC Nyquist SK Maiello P Chao M Borish HJ . CD4+ T cells re-wire granuloma cellularity and regulatory networks to promote immunomodulation following Mtb reinfection. Immunity. (2025) 58:513–4. doi: 10.1016/j.immuni.2025.01.001, 39818207 PMC11832191

[29] Ayasoufi K Wolf DM Namen SL Jin F Tritz ZP Pfaller CK . Brain resident memory T cells rapidly expand and initiate neuroinflammatory responses following CNS viral infection. Brain Behav Immun. (2023) 112:51–76. doi: 10.1016/j.bbi.2023.05.009, 37236326 PMC10527492

[30] Gilpin TE Walter FR Herbath M Sandor M Fabry Z. *Mycobacterium bovis* Bacillus Calmette-Guérin-infected dendritic cells induce TNF-α-dependent cell cluster formation that promotes bacterial dissemination through an *in vitro* model of the blood-brain barrier. J Immunol. (2021) 207:1065–77. doi: 10.4049/jimmunol.2001094 34321229 PMC8592275

[31] Barnacle JR Davis AG Wilkinson RJ. Recent advances in understanding the human host immune response in tuberculous meningitis. Front Immunol. (2023) 14:1326651. doi: 10.3389/fimmu.2023.1326651 38264653 PMC10803428

[32] Kalita J Tripathi A Shukla R Misra UK Kumar S. Role of caspase- 3, TNF-alpha, and IL6 mrna expression in intracranial tuberculoma. Mol Neurobiol. (2022) 59:4869–78. doi: 10.1007/s12035-022-02901-8 35654994

